# Attachment and Political Personality are Heritable and Distinct Systems, and Both Share Genetics with Interpersonal Trust and Altruism

**DOI:** 10.1007/s10519-024-10185-y

**Published:** 2024-05-30

**Authors:** Thomas Haarklau Kleppesto, Nikolai Olavi Czajkowski, Olav Vassend, Espen Roysamb, Nikolai Haahjem Eftedal, Jennifer Sheehy-Skeffington, Eivind Ystrom, Jonas R. Kunst, Line C. Gjerde, Lotte Thomsen

**Affiliations:** 1 Centre for Fertility and Health, Norwegian Institute of Public Health, Oslo, Norway; 2 PROMENTA Research Center, Department of Psychology, University of Oslo, Oslo, Norway; 3 Department of Psychology, Norwegian University of Science and Technology, Trondheim, Norway; 4 Division for Mental and Physical Health, Norwegian Institute of Public Health, Oslo, Norway; 5 Department of Psychology, University of Oslo, Oslo, Norway; 6 Department of Psychological and Behavioural Science, London School of Economics and Political Science, London, UK; 7 Center for the Experimental Philosophical Investigation of Discrimination, Department of Political Science, Aarhus University, Aarhus, Denmark

**Keywords:** Political personality, Politics, Ideology, Attachment, Altruism, Trust, Social dominance orientation, Right-wing authoritarianism, Genetics

## Abstract

The attachment and caregiving domains maintain proximity and care-giving behavior between parents and offspring, in a way that has been argued to shape people’s mental models of how relationships work, resulting in secure, anxious or avoidant interpersonal styles in adulthood. Several theorists have suggested that the attachment system is closely connected to orientations and behaviors in social and political domains, which should be grounded in the same set of familial experiences as are the different attachment styles. We use a sample of Norwegian twins (N = 1987) to assess the genetic and environmental relationship between attachment, trust, altruism, right-wing authoritarianism (RWA), and social dominance orientation (SDO). Results indicate no shared environmental overlap between attachment and ideology, nor even between the attachment styles or between the ideological traits, challenging conventional wisdom in developmental, social, and political psychology. Rather, evidence supports two functionally distinct systems, one for navigating intimate relationships (attachment) and one for navigating social hierarchies (RWA/SDO), with genetic overlap between traits within each system, and two distinct genetic linkages to trust and altruism. This is counter-posed to theoretical perspectives that link attachment, ideology, and interpersonal orientations through early relational experiences.

**Supplementary Information:**

The online version contains supplementary material available at 10.1007/s10519-024-10185-y.

Two evolved hallmarks of mammalian group-living species are the attachment system, which sustains parental offspring care, and dominance hierarchies, which coordinate the distribution of scarce resources and rights (Boehm 1999; Bowlby 1969; Simpson and Belsky 2008; Van Vugt and Tybur 2015). The attachment system evolved to motivate infants to seek proximity and solicit help from their caregivers when needed, and parents, in turn, are motivated by their caregiving system to provide care (Ainsworth 1989; Bowlby 1969; Geary and Bjorklund 2000). The evidence for this function is relatively clear in humans and many other mammals with offspring that cannot fend for themselves (see Fraley et al. 2005; Szepsenwol and Simpson 2019). However, another proposed function of attachment in humans is that these early socialization experiences lead to the development of interpersonal working models, that will in turn extend to broad (secure, anxious or avoidant) attachment patterns between romantic partners and friends in adulthood (Fraley and Shaver 2000, 2021; Hazan and Shaver 1987). Because these working models are thought to specify generalized expectations for help and protection in relationships, attachment styles have been argued to extend to generalized interpersonal trust (the belief that others can be trusted to be helpful) (Mikulincer 1998; Simpson 2007), and altruism (paying a cost to help others) (Gillath et al. 2005; Mikulincer and Shaver 2005; Mikulincer et al. 2005), and even the development of political attitudes (Boag and Carnelley 2016; Green and Douglas 2018; Koleva and Rip 2009; Roccato et al. 2013; but see also Thornhill and Fincher 2007; Weber and Federico 2007).

Such an account of the underlying predictive power of early attachment-related experiences has implications for the association between attachment styles, interpersonal orientations such as trust and altruism, and ideological variables, implying that they should be grounded in childrearing and other family experiences (see Duckitt 2001). Alternatively, recent work that positions political attitudes and the ideological orientations underpinning them as the manifestation of dedicated mechanisms evolved for navigating dominance hierarchies (see Kleppesto et al. 2024; Sheehy-Skeffington and Thomsen 2020; Sidanius and Pratto 1999) would expect ideology to exhibit a genetically-grounded correlation with trust and altruism that is independent of attachment, and that any association between ideology and attachment style be genetic, as opposed to environmental, in origin.

Sex differences in political traits are well-documented (see McDonald et al. 2012). It is therefore crucial to investigate if there are any sex differences in how genetic and environmental factors impact variation in political and social traits.

The following study applies a genetically informative design to address these questions that lie at the heart of social and personality psychology.

## The hypothesized role of early experiences for attachment and ideological orientations

A long-disputed subject in the social sciences concerns whether people’s socioemotional development influences political attitudes. Fromm (1942) and Adorno and colleagues (1950) asserted that a harsh and strict parenting style can lead to the development of antidemocratic tendencies, such as the preference for strong-man leaders and a desire for punishing perceived deviants such as homosexuals, Jews, and radical intellectuals. Similarly, Miller (1980) even argued that authoritarian and destructive tendencies among adults are primarily the result of a childhood characterized by parental violence.

Authoritarian and xenophobic character traits matter as contemporary research has shown how they play an important role in the development of people’s political attitudes (Huddy et al. 2013). Specifically, the degree to which people prefer a traditionalist and authoritarian societal structure, measured by right-wing authoritarianism (RWA) as well as the degree to which they prefer a hierarchical intergroup structure, known as social dominance orientation (SDO) have emerged as central predictors of policy attitudes, prejudice, ethnocentrism, nationalism, support for the far-right, and even willingness to participate in violently persecuting out-groups (Altemeyer 1988; Duckitt 2001; Duckitt and Sibley 2010; Kleppesto et al. 2020; Pratto et al. 1994; Sidanius et al. 2016; Sidanius and Pratto 1999; Thomsen et al. 2008).

A long research tradition has provided evidence consistent with the general view that harsh parenting and childhood rearing experiences affect adult levels of authoritarianism, although they lack control for genetic confounding. For example, perception of parental control was associated with RWA (Heydari et al. 2013), and authoritarianism in parents is correlated with their children reporting a punitive parenting style that had a negative influence on their parent–child relationship (Peterson et al. 1997).

The dual process motivational (DPM) model of ideology (Duckitt 2001; Duckitt and Sibley 2010) argues that early socialization experiences directly influence both RWA and SDO (Duckitt 2001; Duckitt and Sibley 2010). Specifically, the dual process model argues that future right-wing authoritarians are generated by rigid and punitive child-rearing practices because such practices lead to the general view of the world as dangerous and threatening. On the other hand, high SDO is said to develop through unaffectionate parenting, nurturing a tough-mindedness and view of the world as a competitive jungle—an amoral struggle for resources and power (Duckitt 2001).

These theoretical views resonate with decades of work on attachment that has documented three contrasting orientations toward intimate relationships. First, anxiously attached people experience a heightened state of arousal and general preoccupation and worry about close relationships, compulsively seeking proximity and protection. Next, avoidantly attached people are uncomfortable with closeness, strive for self-reliance, and emotionally distance themselves in close relationships (Hazan and Shaver 1987; Mikulincer and Shaver 2003, 2005). Securely attached individuals are characterized by both low avoidance and anxious attachment. Hence, they are generally comfortable with close and intimate relationships, while also being tolerant and appreciative of some autonomy and distance.

One account why such attachment variation exists is that an attachment system which allows for variability in approaches to intimate relationships depending on environmental input provides a crucial fitness advantage (Simpson and Belsky 2008; Szepsenwol and Simpson 2019).

In sum, the literature on the origins both of ideological attitudes and of attachment styles point to an important role for early childhood experiences, with prominent models explicitly linking the two. By this account, a primary factor driving variation in both political traits and interpersonal traits is the common set of societal and relational experiences to which parents, children, and their siblings are exposed.

## Interpersonal trust and altruism as a bridge between attachment and ideology

Because working models for attachment in close relationships are thought to specify generalized expectations for help and protection in other relationships as well, attachment styles have been argued to extend to generalized interpersonal trust, defined as the belief that others can be trusted to be helpful (Mikulincer 1998; Simpson 2007), and altruism, defined as paying a cost to help others (Gillath et al. 2005; Mikulincer and Shaver 2005; Mikulincer et al. 2005).

For example, people scoring high on avoidance and anxiety were less likely to care for someone who had been diagnosed with cancer (Westmaas and Silver 2001). Conversely, Mikulincer and Shaver (2001) found that contextual activation of attachment security led people to be less negative towards out-group members. Hence, Mikulincer and Shaver (2005) argue that attachment security provides a foundation for compassion and caregiving behavior, while anxious and avoidant attachment interferes with these systems. Indeed, people with a secure (versus insecure) relationship style are more likely to have a sense of self-confidence and empathy, and a perception that the world is safe and consists of people one can trust (Mikulincer and Shaver 2003, 2005).

Research on the relationships between attachment style and political ideology/personality, as captured by SDO and RWA, has been conflicting: While Weber and Federico (2007) found that avoidant attachment was related to higher SDO and anxious attachment related to higher RWA, Thornhill and Fincher (2007) found that secure attachment was related to conservatism, but did not find any significant relationships of attachment with RWA and SDO. Others have found that avoidant attachment relates positively to SDO and negatively to RWA and that anxious attachment relates to neither (Roccato et al. 2013; Sinn and Hayes 2018), and others still have found that both avoidant and anxious attachment relate positively to SDO but not to RWA (Green and Douglas 2018).

In sum, the generally accepted view seems to be that early socio-emotional experiences shape attachment style, which has downstream effects both on tendencies to trust and help unknown others, and on preferences for societies to be organized in a way that guards against danger and competition arising from others. Evidence for the associations between these variables is not clear cut, however, and such standard phenotypic correlational studies cannot directly assess the role of early childhood experiences as opposed to other sources of variability.

## Investigating the genetic and environmental grounding of attachment, SDO, and RWA

Much classic work on the importance of early childhood conditions in the development of attachment, trust, altruism and ideology depends on observations of the association between parents and children (Adorno et al. 1950; Ainsworth 1989; Bowlby 1969; Duckitt 2001; Fraley and Shaver 2000; Mikulincer and Shaver 2003). The onset of behavioral genetics methods, however, has highlighted how much of what appears to reflect familial experiences may in fact reflect commonalities in the set of genes shared by family members (see Hart et al. 2021). Through studying identical (monozygotic (MZ)) and fraternal (dizygotic (DZ)) twins who share all or half of their genotypes, respectively, variation in traits and behaviors can be partitioned into genetic factors (A), environmental factors having equal effect on siblings in the same family (i.e. the “shared environment”, or C), and environmental factors unique to each sibling in a family (i.e. the “unique environment”, or E). In the section below, we review evidence from such genetically informed research designs on the social and political variables we investigate in this study.

### Behavioral genetic studies of attachment, altruism, and trust

The largest behavioral genetic study on adult attachment found that the shared environment did not play a role at all for either anxious or avoidant attachment in adults, and that both dimensions had heritabilities of 45% and 39%, respectively (Donnellan et al. 2008). The same supportive and responsive parenting styles that are presumed to lead to secure attachment are also those argued to underpin altruism and general prosocial behavior (Heydari et al. 2013; Peterson et al. 1997). Yet here, too, genetically informative research has generally found that altruism has substantial heritability and that this heritability tends to increase with age (Knafo and Israel 2010; Knafo et al. 2008; Rushton 2004). Similarly, interpersonal trust has also been identified as a highly heritable trait (Cesarini et al. 2008; Sturgis et al. 2010).

### Behavioral genetic studies of ideology

The application of the twin method to the study of political attitudes has led to accumulating evidence for substantial heritability in ideological preferences amongst adults (Alford et al. 2005; Bouchard and McGue 2003; Dawes and Weinschenk 2020; Hatemi et al. 2014; Jang et al. 1996; Morosoli et al. 2022; Settle et al. 2009). Research is now moving from a focus on political attitudes and left–right voting to the study of the origins of variation in underlying ideological orientations concerning inequality and authority, as measured by SDO (Sidanius and Pratto 1999) and RWA (Altemeyer 1988; Zakrisson 2005), respectively. Behavioral genetic evidence suggests that both SDO and RWA is moderately heritable (de Vries et al. 2022; Kandler 2015; Kandler et al. 2016; Kleppesto et al. 2019; Ludeke and Krueger 2013). The very few multivariate behavioral genetics studies that have been conducted in this area suggest that the covariance between basic hierarchy-related traits and political preferences are due to a common, latent genetic factor that mutually influences both (Kleppesto et al. 2019; Lewis and Bates 2014; Verhulst et al. 2012). This has also been shown with emotional traits and political policies: The correlation between social fear and negative views of out-groups are best explained by shared genetic mechanisms (Hatemi et al. 2013).

## The present study

It has been suggested that childrearing practices emphasizing beliefs that the world is fundamentally dangerous should generate high RWA and anxious attachment, while childrearing practices associated with the perception that the world is an uncaring, competitive-jungle generates high SDO, and also avoidant attachment (Duckitt 2001; Weber and Federico 2007). Such an account of a single relational system would predict a shared environmental correlation between these sets of variables and interpersonal orientations such as altruism and trust. Corroboration of these theoretically based phenotypic relationships between attachment, trust, altruism, and the political traits have so far been mixed. We therefore intend to explore their relationships with a large sample, in addition to decompose the covariation between them into genetic and environmental components, which can shed light on their etiology.

With the data we have on hand here, we would also like to investigate a specific account that presumes *distinct* adaptive systems for intimate relationships and hierarchy navigation. If these two systems are distinct, one would expect that the two attachment domains show overlap in their genetic structure, that SDO and RWA would show overlap in their genetic structure, but no overlap between attachment and political ideology. Hence, if both attachment style and ideological orientations predict altruism and trust, it would be for different reasons, thus explaining different components of the variance in these interpersonal orientations.

Here, we take advantage of a large sample twin design to test these competing proposals. To our knowledge, the genetic and environmental relationship between RWA and SDO, on one hand, and attachment, trust and altruism on the other, has not been investigated. It is also necessary to test for potential differences across sex in genetic and environmental effects. We therefore include models that test for the possibility that the impact from genetic and environmental factors on trait variation can differ between the sexes (so-called ‘sex limitation’ models, see Neale et al. 2006). Thus, genetically informative studies on adults are needed to elucidate the genetic and environmental etiology of attachment, trust, altruism and political traits, and to shed light on whether they are all part of the same system grounded in early life experiences, or are instead independent social domains grounded in genetic differences.

## Methods

### Sample

The sample consisted of twins recruited via the Norwegian twin registry (NTR), which consists of several cohorts of twins (Nilsen et al. 2013). The cohort used here consists only of randomly drawn same-sex twins born between 1945 and 1960. The mean age of the whole sample was 65.16 (*SD* = 4.49; range 56–71). The measurements were made in 2016 and we had 708 complete twin pairs responding, as well as 571 additional single responders (total response rate was 64%). For sample sizes and descriptives across zygosity and sex, see Table A1. We determined zygosity with a questionnaire that has been shown to correctly classify 97% of twins (Magnus et al. 1983).

### Measures

#### Social dominance orientation (SDO)

The participants completed a Norwegian translation of the SDO-7 scale (Ho et al. 2015). It consists of 16 items rated on a 7-point Likert scale ranging from 1 (*strongly oppose*) to 7 (*strongly favor*), such as “Some groups of people must be kept in their place.” Due to methodological warnings that respondents might not reliably process and respond to negative-worded items (Roszkowski and Soven 2010), all SDO-7 items were administered protrait on their respective subdimension (for group dominance or for group equality, respectively), with the equality items reverse-coded in the computing of the SDO composite. Cronbach’s α for the scale was 0.85.

#### Right-Wing Authoritarianism (RWA)

The participants completed a 15-item version of RWA (Zakrisson 2005). The items were rated on 7-point Likert scales ranging from 1 (*strongly disagree*) to 7 (*strongly agree*). An example is “The old-fashioned values still show the best way to live”. Cronbach’s α for the scale was 0.75.

#### Attachment

Anxious and avoidant attachment patterns were measured with a Norwegian translation of the Experiences in Close Relationships scale (ECR-12) using 7-point Likert scales ranging from 1 (*strongly disagree*) to 7 (*strongly agree*) (Brennan et al. 1998; Olssøn et al. 2010). The items that measure anxious and avoidant attachment styles were determined according to the two-factor structure reported by Olssøn and colleges (2010). Examples include “I worry about being abandoned” (anxiety) and “I try to avoid getting too close” (avoidant). Cronbach’s α for the scales was 0.84 for avoidant, and 0.77 for anxious.

#### Trust

We used a measure of generalized trust from the European Social Survey (European Social Survey Data Archive 2018). The scale consists of three items rated on an 11-point scale from 0 to 10. Higher scores means higher trust. Examples include, “Would you say that most people can be trusted, or that you can’t be too careful in dealing with people?”, and “Do you think that most people would try to take advantage of you if they got the chance, or would they try to be fair?”. Cronbach’s α for the scale was 0.82.

#### Altruism

Altruism was measured with a 5-item version of the Self-report Altruism Scale (Rushton et al. 1981). The scale includes five items measuring different altruistic behaviors on a 5-point scale, ranging from “never”, to “very often”. Examples include, “I have donated blood”, and “I have given money to a stranger who needed it (or asked me for it)”. Cronbach’s α for the scale was 0.42.

### Analyses

The classical twin method makes it possible to partition the variation of any trait into three components A, C and E by investigating patterns of correlation between MZ twins and DZ twins (Neale and Maes 2004). A denotes additive genetic influences; C denotes shared environmental influences, that is, environmental influences that make twin pairs more similar; E denotes unique environmental influences, that is, environmental influences that make twin pairs less similar. As MZ twins share all their genetic material, and DZ twins share on average half of segregating genes, any increased similarity within MZ pairs compared to DZ pairs can be attributed to A influences.

Structural equation modelling with Cholesky decomposition was used in order to partition and model both the variances and covariances of the twins in terms of additive genetics (A), shared environment (C), and unique environment (E). We first ran all our models with four variables (anxious attachment, avoidant attachment, RWA and SDO), to then use the best-fitting model as the basis for further analysis with trust and altruism included. The model that formed the basis of our estimates of environmental (rE) and genetic (rA) correlations came from the best-fit Cholesky model with all six variables included and a shared environmental effect estimated only for RWA (see below for details).

We tested ten models in total (see Table 3). We selected these models to investigate if the three variance components (A, C and E) were all necessary to account for the data, or if more parsimonious models were sufficient, and also to investigate if there were any differences across sex in the contribution to the variance components. Multivariate ACE models allow us to estimate genetic and environmental correlations between the phenotypes. Such correlations estimate to what extent the variances in the traits are due to common genetic (rA) or common environmental (rE) causes. For example, if a genetic correlation between two heritable traits is zero, it means that the genetic effects on one phenotype are independent of those on the other. If it is one, it means that the genetic effects on one trait are completely overlapping with the other. This is also the case with environmental correlations. For example, a unique environmental correlation (rE) of zero between two traits means that all unique environmental influences on one phenotype are independent of the influences on the other phenotype. A unique environmental correlation of one, on the other hand, means that the unique environmental influences completely overlap.

We first tested a full model, in which we allowed for the influence of A, C, and E on all traits and quantitative sex differences (model 1). Quantitative sex differences imply that, while the same genetic and environmental factors influence the phenotypes in males and females, they may do so to a different extent. This is modelled by the estimation of separate path loadings for males and females, but constraining the genetic (rA), shared environmental (rC), and unique environmental (rE) correlation matrices to be equal across sex (see Neale et al. 2006). In a subsequent set of models (2–6, labeled “prop eq” in Table 3), in addition to constraining the correlation, the proportion of variance that could be attributed to A, C and E was also constrained to be equal across sex, while allowing the overall variance to differ. In these models, the heritability of anxious attachment, for example, can be equal in males and females, but the trait could have a higher phenotypic variance in females. Univariate estimates of A, C, and E (see Table 2) for every variable, suggested the presence of a strong and statistically significant C effect for RWA, and smaller C effects for attachment. We therefore included models with one general C effect for all four variables, and a model with a C effect specific for RWA (model 3 and 4, respectively). For models five and six we set the C and the A parameters to zero, respectively, in order to check whether these more parsimonious models were sufficient.

In the last set of models (7–10), path coefficients as well as phenotypic variances were constrained to be equal across sex. This means that the scalar that tests for different variances across the sexes are not allowed to vary. We never constrained E to zero, because it contains all the residual variance not due to A and C, and hence also includes measurement error in the phenotypes.

All model parameters were estimated using the R package OpenMx (Neale et al. 2016). We used Akaike’s Information Criterion (AIC) to select the overall best fitting model (Akaike 1987). Low AIC indicates better model fit.

### Results

The phenotypic correlations were generally very low between attachment and the hierarchy-related traits, with the highest correlation being 0.11 between anxious attachment and RWA, and 0.07 between avoidant attachment and SDO (see Table 1). Anxious and avoidant attachment, as well as RWA and SDO all have negative phenotypic correlations with trust (ranging from − 0.20 to − 0.23), as well as in smaller magnitude for altruism. For descriptive statistics of anxious attachment, avoidant attachment, SDO, RWA, trust and altruism, see Table A1.

**Table 1:** Phenotypic correlations (with 95% CI)

Variable	Anxious	Avoidant	RWA	SDO	Trust
Avoidant	.45				
	(.39, .50)				
RWA	.11	.06			
	(.04, .17)	(− .01, .12)			
SDO	.04	.07	.30		
	(− .03, .11)	(.01, .14)	(.24, .36)		
Trust	− .20	− .20	− .22	− .23	
	(− .27, − .14)	(− .26, − .14)	(− .28, − .16)	(− .29, − .17)	
Altruism	− .08	− .12	− .15	− .07	.13
	(− .15, − .02)	(− .18, − .05)	(− .21, − .09)	(− .14, − .01)	(.06, .19)

*RWA* Right-Wing Authoritarianism, *SDO* social dominance orientation

## Twin correlations and univariate estimates

The twin correlations (see appendix, Table A2–A5) indicated that MZ twins correlated more highly than DZ twins for all variables, suggesting genetic influences on the measures. Moreover, MZ and DZ twins have more similar correlations for RWA, especially for females, suggesting a C effect (C effects are implied when rDZ is more than half of rMZ). Estimates of the A, C, and E parameters indeed show similar heritabilities for all variables (ranging from 0.22 to 0.32), with RWA specifically showing a significant C effect (see Table 2).

**Table 2:** ACE estimates for every variable from a full ACE model (additive genetic, shared environmental, unique environmental)

	Variance components (95% CI)		
	A (contribution of heritability)	C (contribution of shared environment)	E (contribution of unique environment)
Anxious	0.22 (0, .44)	0.14 (0, .35)	0.64 (.55, .74)
Avoidant	0.23 (.05, .4)	0.13 (0, .29)	0.65 (.56, .74)
RWA	0.32 (.16, .5)	0.30 (.15, .44)	0.37 (.32, .43)
SDO	0.30 (.13, .4)	0.02 (0, .16)	0.68 (.6, .76)
Trust	0.25 (.06, .4)	0.07 (0, .22)	0.68 (.6, .77)
Altruism	0.32 (.11, .46)	0.08 (0, .26)	0.6 (.52, .69)

*RWA* Right-Wing Authoritarianism, *SDO* social dominance orientation

## Multivariate genetic modelling

**Fig. 1:**
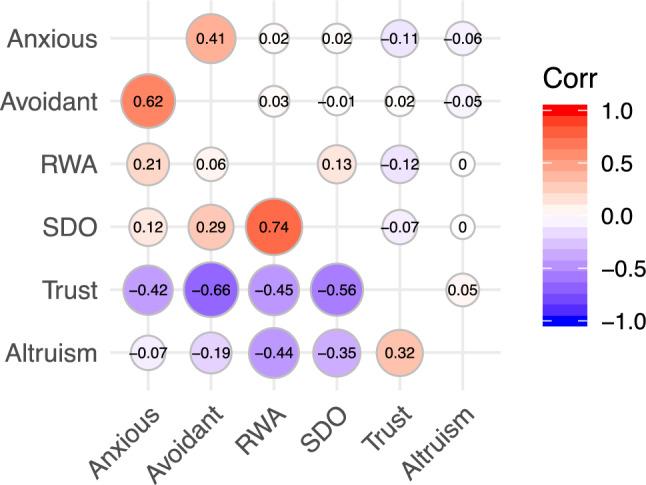
Genetic (below diagonal) and unique environmental (above diagonal) correlation matrix. *RWA* Right-Wing Authoritarianism, *SDO* Social Dominance Orientation. A genetic correlation of 1 means that all the genetic variance is shared between two variables. A unique environmental correlation of 1 means that all unique environmental influences are shared between the traits. The genetic correlations are below the diagonal, while the unique environmental correlations are above the diagonal. See Table A6 for confidence intervals

Our best fitting model was an AE Cholesky model without sex limitation, with one unique C factor with moderate loading on RWA (Model 4 in Table 3). This model was run with anxious attachment, avoidant attachment, RWA and SDO. This aligns with the univariate modelling of the variables, where RWA was the only variable with a substantive C effect (where the confidence intervals did not include zero), hence the AIC fit index rewards this more parsimonious model, compared to a model where C is modelled for every variable (model 3) or not at all (model 5). We used this best-fitting model as the basis when deriving estimates with trust and altruism included. The genetic and unique environmental correlations between all variables are shown in Fig. 1.

**Table 3:** Model fit statistics for multivariate model of anxious and avoidant attachment, right wing authoritarianism and social dominance orientation

Model	EP	− 2LL	Df	Δ − 2LL	Δ df	AIC	p
(1) SL Cholesky ACE	68	19,333.59	7,284	NA	NA	4,765.587	NA
(2) NS Cholesky ACE (prop eq.)	42	19,354.41	7,292	20.823	8	4,770.411	.008
(3) NS AE1C (prop eq.)	36	19,355.80	7,298	22.208	14	4,759.796	.074
**(4) NS AEUC (prop eq.)**	**33**	**19,360.24**	**7,301**	**26.657**	**17**	**4,758.245**	**.063**
(5) NS AE (prop eq.)	32	19,373.95	7,302	40.365	18	4,769.953	.002
(6) NS CE (prop eq.)	32	19,384.21	7,302	50.618	18	4,780.206	< .001
(7) NS ACE	38	19,367.22	7,296	33.633	12	4,775.220	.001
(8) NS AE1C	32	19,368.64	7,302	35.056	18	4,764.643	.009
(9) NS AE	28	19,386.26	7,306	52.669	22	4,774.256	< .001
(10) NS CE	28	19,398.12	7,306	64.531	22	4,786.119	< .001

*Note.* *EP* number of parameters, *LL*  LogLikelihood, *df* degrees of freedom; Δ difference to base model, *AIC* Akaike’s information criterion, *p*  p-values from test of difference from base model. Best-fitting model indicated in bold. *SL* sex limitation, *NS* no sex limitation, *A* additive genetics, *C* shared environment (1C meaning one C factor that loads on all variables, UC one unique C factor for RWA), and *E* unique environment, *Prop eq.*  proportions equal, meaning that all the standardized path coefficients are equal across sex, but the phenotypic variances across sex are allowed to be different. In models 7–10 (without prop. eq.) both the phenotypic variances across sex, as well as the proportions attributable to A, C and E, are constrained to be equal

RWA and SDO correlated phenotypically (rP = 0.30) and the variance that they share was largely due to genetic factors (rA = 0.74). Avoidant and anxious attachment were phenotypically correlated at 0.45, and their genetic correlation was 0.62.

The genetic correlations between the hierarchy-related variables (RWA and SDO) and attachment reveal that RWA is mostly genetically correlated with anxious attachment, while SDO is mostly genetically correlated with avoidant attachment, and such genetic correlations were higher than unique environmental correlations, which were close to zero. This implies that the very small phenotypic correlations between the hierarchy-related variables and attachment are primarily genetic in origin. Note that it can be important to investigate genetic and environmental correlations even when the phenotypic correlation is low, or even zero. This is because genetic and environmental correlations can go in opposite directions and therefore generate a low phenotypic correlation (Falconer and Mackay 1996). Nevertheless, see Figure A1 and A2 in the appendix for an illustration of the very small amount of shared genetic (and environmental) variance between the two attachment variables and RWA (Figure A1), and between the two attachment variables and SDO (Figure A2).

Although the hierarchy-related traits and attachment share very little variance both phenotypically and etiologically with each other, they both reveal separate consistent negative relationships with interpersonal trust. The phenotypic relationships between insecure attachment and trust, and the hierarchy-related traits and trust, are very similar, ranging from − 0.20 to − 0.23 (see Table 1). In the case of both attachment and hierarchy-related traits, these associations are primarily genetic in origin, as shown below the diagonal in Fig. 1. Thus, although attachment and hierarchy-related traits are largely decoupled from each other, they both have an independent, genetically-grounded relationship with interpersonal trust. A similar pattern also reveals itself with altruism, though here the genetic correlations are stronger for the hierarchy-related traits (− 0.35 for SDO and − 0.44 for RWA) than for anxious (− 0.07) or avoidant (− 0.19) attachment style, and the phenotypic correlations are overall smaller (ranging from − 0.07 to − 0.15).

## Discussion

With a large-sample twin design, this study provides the first evidence that regulating intimate relationships (attachment) and regulating social hierarchies (authoritarianism and SDO) are largely unrelated both at the phenotypical and etiological level. Insecure attachment and the hierarchy-related traits both provided moderate negative contributions to interpersonal trust, and weak negative contributions to altruism at the phenotypic level. All of these relationships were due to overlap in genetic variation, rather than environmental overlap. There were no sex differences in the impact of the genetic and environmental components underlying the traits.

Most theorizing on political traits, attachment, trust and altruism have proposed environmental explanations for their variation and covariation. For example, Thornhill and Fincher asked themselves if the behaviors associated with politics are byproducts of attachment adaptations. They hypothesized that “individual differences in political values are manifestations of species-typical psychological adaptation of attachment, which in turn ontogenetically arises from experiences of early childhood stressors” (2007, p. 216). This hypothesis is also congruent with Fromm (1942) and Adorno et al.’s (1950) idea that authoritarian values are generated from negative parenting experiences, in addition to claims in political psychology that both RWA and SDO stem from early socialization (Duckitt 2001; Duckitt and Sibley 2010). Some studies have found an association between attachment styles and the development of political ideologies, trust, and altruism. Other studies have not. The inconsistent empirical data has likely been due to low sample sizes in most studies investigating these relationships. Past research has also been on genetically insensitive samples, making controls for genetic confounding impossible.

Using a genetically informative design, we investigated central predictions that stem from an account in which ideological orientations are grounded in attachment styles: that there are substantial phenotypic correlations between adult attachment and RWA and SDO, that each of them show evidence of shared environmental variance, and that this shared environmental variance will correlate across the traits. We do not find evidence consistent with any of these conjectures. Instead, the data presented here are consistent with an account of two distinct heritable systems.

We find that attachment is not in any significant way explained by the shared environment of twins in this study. This finding runs contrary to the postulations of attachment theory, which suggests that variance in attachment patterns calibrates within the family and last into adulthood (Fraley and Shaver 2021). For example, in an integrative review of behavior genetics and attachment theory, it was argued that what is novel about attachment theory as a developmental socioemotional theory is that it makes “a strong prediction that [a shared environment effect, as revealed through] the concordance between MZ and DZ twins will be similar and substantial” (O’Connor et al. 2000, p. 111).

Our results are in line with the largest behavioral genetic study on adult attachment, which finds no evidence of shared environment effects (Donnellan et al. 2008). It is possible that attachment in children and in adults are different constructs, and that this is the reason why shared environment effects of attachment are measurable in infants, but not typically in adults, including in this study. However, what has helped to make attachment theory so influential is the prediction that insecurely attached children will develop working models about relationships that last into adulthood (Fraley and Shaver 2000, 2021; Hazan and Shaver 1987). Recent longitudinal attachment studies have suggested that attachment is not as stable as once thought (see Fraley and Roisman 2019). One key advantage with the current study is that the participants are middle-aged adults, and hence we can capture any long-lasting effects of family environments captured by the shared environment (C). If attachment has evolved to “set” itself during a developmental period in infancy, the shared environment effect of attachment in children should be detectable in adulthood. The data in this paper is inconsistent with such an idea. Nevertheless, genetically sensitive longitudinal studies that measure how the relationships between attachment, trust, altruism, and ideology might change over time from childhood to old age would be illuminating.

Anxious and avoidant attachment styles were correlated phenotypically (*r* = 0.45), underpinned by both genetic (rA = 0.62) and unique environmental (rE = 0.41) components. The unique environmental correlation suggests that the differentiation into secure, anxious or avoidant attachment is driven largely individual-specific non-genetic processes, rather than shared (or within-family) experiences.

We also find that two of the most influential ideological orientations in social psychology are each substantially genetically grounded, and intriguingly, share their genetic substrate to a large degree. That is, we find that RWA and SDO are strongly genetically related (rA = 0.74), suggesting that they may form part of the same evolved system for navigating social hierarchy (Sheehy-Skeffington and Thomsen 2020; Thomsen et al. 2008), and is consistent with the high genetic correlations reported elsewhere (Nacke and Riemann 2023). RWA and SDO have been found to share strong predictive power when it comes to the same political attitudes (e.g., prejudice, nationalism, and right-wing politics), leading to their integration in the influential dual process model of ideology and prejudice (DPM, see Duckitt 2001; Duckitt and Sibley 2010). Indeed, previous behavioral genetics research on SDO and RWA reported that they both share the underlying common factor of support for aggression against low power groups (Kandler et al. 2016). Yet core to the dual process model is the claim that early childhood experiences in the home, through affecting views of the world as dangerous and/or competitive, set levels of RWA and SDO (respectively), a shared environmental effect that we find only for RWA. Our finding that RWA and SDO are strongly genetically related (rA = 0.74) suggests that they form part of the same evolved system for navigating social hierarchy, but might be mobilized differently depending on whether the focus is within- (RWA) versus between- (SDO) group regulation (Nacke and Riemann 2023; Sheehy-Skeffington and Thomsen 2020; Thomsen et al. 2008).

For RWA, note that the differences in correlations between MZ and DZ twins are substantially more pronounced in males than females, as indicated by Tables A2–A5. This disparity apparently reveals a female-only shared environmental effect. Future studies should aim not only to replicate this finding, but also to explore the underlying mechanisms responsible for a shared environmental effect on RWA, with a focus on the potential differentiation by sex.

Despite the genetic overlap within the attachment system and within what we deem to be a system for navigating social hierarchy (RWA and SDO), there is very little overlap between the two systems. Phenotypic correlation coefficients were very low, with the highest occurring between anxious attachment and RWA (r = 0.11), and between avoidant attachment and SDO (r = 0.07). This weak pattern of differential pairing of attachment styles and ideological orientations was mirrored when we looked at the genetic underpinnings. RWA has a 0.21 genetic correlation with anxious attachment and 0.06 with avoidant, while SDO has 0.12 genetic correlation with anxious attachment, but 0.29 with avoidant. These differential relationships are in line with Weber and Federico’s (2007) predictions, but should be interpreted with caution given the low phenotypic relationships between attachment and the ideological orientations.

The functional distinctness of the attachment and hierarchy navigation systems does not preclude their both having an influence on interpersonal orientations. Indeed, we do find evidence that trust, and to some degree altruism, is negatively genetically correlated with both attachment and the ideological orientations, even though attachment and the hierarchy-related traits are largely unrelated to each other. Individuals who are concerned with the maintenance of social hierarchy (those high in RWA and/or SDO) have been found to perceive others as threatening to their well-being (Perry et al. 2013), a worldview intuitively linked to low levels of trust. Although the phenotypic correlations involving altruism are lower, it is notable that the hierarchy-related traits have stronger genetic correlations with altruism (rA = − 0.35 for SDO, rA = − 0.44 for RWA) than do the attachment styles (rA = − 0.07 for anxious and rA = − 0.19 for avoidant). This suggests that there might be an evolved functional linkage between concern for hierarchy and willingness to share resources, as suggested by emerging developmental and political psychology research (see Sheehy-Skeffington and Thomsen 2020).

Sinn and Hayes’ (2018) have conjectured that RWA is a prosocial adaptation and SDO is an antisocial strategy. Contrary to this suggestion we find negative genetic correlations between SDO/RWA and altruism, suggesting that SDO/RWA converge in their downstream impact on social behavior. Individuals with insecure attachment patterns also have low levels of trust in others, and to some degree also are less altruistic, but for different reasons than someone with high RWA and/or SDO. Instead of being motivated by hierarchical zero-sum worldviews, insecurely attached individuals might have lower trust and altruism through a socioemotional pathway, based on strategies for navigating intimate relationships that draw on their own set of heritable influences.

Our study has some important limitations. The altruism scale we used in this paper had low reliability (Cronbach’s α = 0.42). We used a short five-item version of the original full scale with 20 items (Rushton et al. 1981). The items ask for very specific behaviors, such as “I have donated blood”, and “I have helped to push a stranger’s car out of the snow”. This is helpful because it captures specific altruistic acts. The downside is that fewer people will have engaged in all or none of these specific altruistic acts, and hence the intra-correlations between the items will be low. We recommend that future studies use the full-scale altruism scale to capture more of the individual differences in altruism. Our Norwegian sample is also culturally homogenous and has a relatively narrow range of socioeconomic status. It is important that these relationships are investigated in other cultures because different cultural contexts can change estimates of heritability and environmental effects (Uchiyama et al. 2021).

We propose that a possible explanation of our results is a distinct system approach in which attachment styles and ideological orientations each evolved to address challenges in the distinct domains of intimate relationships and hierarchy navigation, respectively, which is consistent with evolutionary domain-specific approaches to intergroup relations (see Buss 1995; Tooby and Cosmides 2015). This is counter-posed to a single system approach (advanced by classic attachment theorists and the dual process model of ideology) that links attachment, ideology, and interpersonal orientations through early relational experiences.

Overall, the result of our study of attachment, trust, altruism and ideology upends much conventional wisdom in the fields of developmental, political, and social psychology. Contrary to popular and long-standing accounts of the causes and consequences of attachment styles, we find no evidence that attachment and ideology are jointly grounded in early familial experiences. The picture that emerges instead is one of two functionally distinct systems, one to navigate intimate relationships (attachment) and the other to navigate social hierarchies (RWA/SDO), both of which matter for how we perceive and treat others in the world (trust and altruism).

### Supplementary Information

Below is the link to the electronic supplementary material.

Supplementary file1 (DOCX 105 KB)

## Data Availability

Access to the data and materials used in this study can be applied for at the Norwegian Twin Registry (NTR).

## Code availability

The code used in this study are unavailable due to ethical and legal constraints given by the Norwegian Twin Registry (NTR).
