# Supplementary material for: Attachment and Political Personality are Heritable and Distinct Systems, and Both Share Genetics with Interpersonal Trust and Altruism
Source: Behav Genet. 2024 May 30;54(4):321–32. doi: 10.1007/s10519-024-10185-y (PMC11196312; doi:10.1007/s10519-024-10185-y)
Supplement: Supplementary file 1 — Supplementary file1 (DOCX 105 KB) [file 10519_2024_10185_MOESM1_ESM.docx]

**Supplementary material for:**

**Attachment and political personality are heritable and distinct systems, and both share genetics with interpersonal trust and altruism**

**Authors:**

Thomas Haarklau Kleppesto (2,3,7) *

Nikolai Olavi Czajkowski (3,4)

Olav Vassend (1)

Espen Roysamb (3,4)

Nikolai Haahjem Eftedal (3)

Jennifer Sheehy-Skeffington (5,6)

Eivind Ystrom (3,4)

Jonas R. Kunst (1)

Line C. Gjerde (3,4)

Lotte Thomsen (1,6)

**Affiliations:**

1. Department of Psychology, University of Oslo, Norway

2. Centre for Fertility and Health, Norwegian Institute of Public Health, Oslo, Norway

3. PROMENTA Research Center, Department of Psychology, University of Oslo, Norway

4. Division for Mental and Physical Health, Norwegian Institute of Public Health, Oslo, Norway.

5. Department of Psychological and Behavioural Science, London School of Economics and Political Science, United Kingdom

6. Center for the Experimental Philosophical Investigation of Discrimination, Department of Political Science, Aarhus University, Denmark

7. Department of Psychology, Norwegian University of Science and Technology, Trondheim, Norway

*** Corresponding author**

**Supplementary figures**

**Figure A1. Genetic and environmental variance of Right Wing Authoritarianism (RWA), with proportions shared and unshared with attachment**

*Note: The vast majority of the phenotypic variation in RWA is due to A, C, and E variance components that is specific to RWA (not shared with attachment). The specific numbers that the figure is based on are given in table S12.*

**Figure A2. Genetic and environmental variance of SDO with proportions shared and unshared with RWA and attachment**

*Note: The vast majority of the phenotypic variation in SDO is due to variance components that is specific to SDO (not shared with attachment). The specific numbers that the figure is based on are given in table S13.*

**Supplementary tables**

**Table A1: Descriptives**

|  | n | | | |  | mean (sd) | | | |
| --- | --- | --- | --- | --- | --- | --- | --- | --- | --- |
|  | MZM | DZM | MZF | DZF |  | MZM | DZM | MZF | DZF |
| Anxious | 348 | 421 | 458 | 540 |  | 2.73 (1.10) | 2.64 (1.07) | 2.68 (1.18) | 2.64 (1.12) |
| Avoidant | 348 | 421 | 458 | 542 |  | 2.32 (1.12) | 2.31 (0.99) | 2.32 (1.08) | 2.31 (1.14) |
| RWA | 357 | 446 | 504 | 588 |  | 3.63 (0.89) | 3.65 (0.84) | 3.76 (0.78) | 3.79 (0.79) |
| SDO | 365 | 448 | 502 | 588 |  | 2.34 (0.94) | 2.44 (0.95) | 2.12 (0.90) | 2.19 (0.89) |
| Trust | 367 | 456 | 523 | 617 |  | 8.27 (1.59) | 8.00 (1.71) | 8.49 (1.65) | 8.39 (1.77) |
| Altruism | 362 | 445 | 511 | 601 |  | 2.90 (0.62) | 2.87 (0.57) | 2.85 (0.62) | 2.84 (0.63) |

*Note: MZM = Monozygotic males; DZM = Dizygotic males; MZF = Monozygotic females; DZF = Dizygotic females; Anxious = Anxious attachment style; Avoidant = Avoidant attachment style; RWA = Right-wing authoritarianism; SDO = Social Dominance Orientation*

**Twin correlations**

**Table A2. MZM twin correlations (with 95% CI)**

| Variable | Anxious | Avoidant | RWA | SDO | Trust | Altruism |
| --- | --- | --- | --- | --- | --- | --- |
|  |  |  |  |  |  |  |
| Anxious | .36 | .24 | .05 | .06 | -.11 | -.18 |
|  | (.20, .51) | (.06, .40) | (-.12, .23) | (-.11, .23) | (-.28, .06) | (-.35, -.01) |
|  |  |  |  |  |  |  |
| Avoidant | .28 | .44 | .04 | .11 | -.19 | -.11 |
|  | (.10, .43) | (.28, .57) | (-.14, .21) | (-.07, .28) | (-.35, -.02) | (-.28, .06) |
|  |  |  |  |  |  |  |
| RWA | .06 | -.09 | .77 | .39 | -.19 | -.29 |
|  | (-.13, .23) | (-.26, .10) | (.68, .83) | (.23, .53) | (-.35, -.01) | (-.45, -.13) |
|  |  |  |  |  |  |  |
| SDO | -.01 | .10 | .32 | .35 | -.18 | -.22 |
|  | (-.19, .17) | (-.08, .27) | (.16, .47) | (.19, .49) | (-.34, -.01) | (-.38, -.06) |
|  |  |  |  |  |  |  |
| Trust | -.24 | -.31 | -.31 | -.21 | .29 | .18 |
|  | (-.40, -.06) | (-.46, -.14) | (-.46, -.14) | (-.37, -.03) | (.13, .44) | (.01, .34) |
|  |  |  |  |  |  |  |
| Altruism | .07 | -.02 | -.08 | -.08 | .02 | .40 |
|  | (-.12, .24) | (-.20, .16) | (-.25, .10) | (-.25, .10) | (-.15, .19) | (.24, .54) |
|  |  |  |  |  |  |  |

*Anxious = Anxious attachment style; Avoidant = Avoidant attachment style; RWA = Right-wing authoritarianism; SDO = Social Dominance Orientation*

**Table A3. DZM twin correlations (with 95% CI)**

| Variable | Anxious | Avoidant | RWA | SDO | Trust | Altruism |
| --- | --- | --- | --- | --- | --- | --- |
|  |  |  |  |  |  |  |
| Anxious | .22 | -.01 | .07 | .04 | -.01 | -.06 |
|  | (.03, .38) | (-.19, .18) | (-.11, .25) | (-.14, .22) | (-.19, .17) | (-.24, .12) |
|  |  |  |  |  |  |  |
| Avoidant | .15 | .15 | .03 | .05 | -.07 | .09 |
|  | (-.03, .33) | (-.04, .32) | (-.15, .21) | (-.13, .23) | (-.25, .11) | (-.10, .26) |
|  |  |  |  |  |  |  |
| RWA | .17 | .08 | .38 | .14 | -.24 | -.03 |
|  | (-.01, .34) | (-.10, .25) | (.22, .52) | (-.04, .30) | (-.40, -.07) | (-.20, .14) |
|  |  |  |  |  |  |  |
| SDO | .20 | .20 | .07 | .05 | -.18 | -.06 |
|  | (.02, .36) | (.03, .37) | (-.10, .24) | (-.12, .22) | (-.34, -.01) | (-.23, .11) |
|  |  |  |  |  |  |  |
| Trust | -.20 | -.13 | -.10 | -.21 | .24 | .23 |
|  | (-.37, -.03) | (-.30, .05) | (-.27, .07) | (-.37, -.04) | (.07, .39) | (.06, .39) |
|  |  |  |  |  |  |  |
| Altruism | -.06 | -.07 | -.11 | -.00 | .06 | .17 |
|  | (-.24, .11) | (-.25, .11) | (-.27, .07) | (-.18, .17) | (-.11, .23) | (-.00, .34) |
|  |  |  |  |  |  |  |

*Anxious = Anxious attachment style; Avoidant = Avoidant attachment style; RWA = Right-wing authoritarianism; SDO = Social Dominance Orientation*

**Table A4. MZF twin correlations (with 95% CI)**

| Variable | Anxious | Avoidant | RWA | SDO | Trust | Altruism |
| --- | --- | --- | --- | --- | --- | --- |
|  |  |  |  |  |  |  |
| Anxious | .38 | .19 | .10 | .05 | -.12 | -.06 |
|  | (.25, .50) | (.04, .33) | (-.05, .24) | (-.10, .19) | (-.25, .03) | (-.20, .08) |
|  |  |  |  |  |  |  |
| Avoidant | .23 | .24 | -.01 | -.06 | -.13 | -.09 |
|  | (.08, .36) | (.09, .37) | (-.16, .13) | (-.20, .09) | (-.27, .01) | (-.23, .06) |
|  |  |  |  |  |  |  |
| RWA | .10 | .10 | .55 | .33 | -.16 | -.12 |
|  | (-.04, .24) | (-.04, .24) | (.44, .64) | (.21, .45) | (-.29, -.02) | (-.25, .01) |
|  |  |  |  |  |  |  |
| SDO | .02 | .13 | .17 | .33 | -.05 | -.04 |
|  | (-.13, .16) | (-.02, .26) | (.03, .30) | (.20, .44) | (-.19, .08) | (-.17, .10) |
|  |  |  |  |  |  |  |
| Trust | -.23 | -.23 | -.18 | -.20 | .32 | .12 |
|  | (-.36, -.09) | (-.36, -.09) | (-.31, -.04) | (-.33, -.07) | (.19, .43) | (-.01, .25) |
|  |  |  |  |  |  |  |
| Altruism | -.02 | -.04 | -.05 | -.19 | .09 | .43 |
|  | (-.17, .12) | (-.18, .11) | (-.18, .09) | (-.32, -.05) | (-.05, .22) | (.31, .53) |
|  |  |  |  |  |  |  |

*Anxious = Anxious attachment style; Avoidant = Avoidant attachment style; RWA = Right-wing authoritarianism; SDO = Social Dominance Orientation*

**Table A5. DZF twin correlations (with 95% CI)**

| Variable | Anxious | Avoidant | RWA | SDO | Trust | Altruism |
| --- | --- | --- | --- | --- | --- | --- |
|  |  |  |  |  |  |  |
| Anxious | .19 | .14 | -.00 | .01 | -.02 | .05 |
|  | (.04, .34) | (-.01, .29) | (-.16, .15) | (-.14, .16) | (-.17, .12) | (-.10, .20) |
|  |  |  |  |  |  |  |
| Avoidant | .11 | .26 | -.01 | .04 | .03 | -.02 |
|  | (-.04, .26) | (.11, .40) | (-.16, .14) | (-.11, .19) | (-.12, .17) | (-.17, .13) |
|  |  |  |  |  |  |  |
| RWA | .03 | .17 | .49 | .04 | -.16 | -.13 |
|  | (-.12, .18) | (.02, .31) | (.38, .60) | (-.10, .18) | (-.30, -.02) | (-.27, .01) |
|  |  |  |  |  |  |  |
| SDO | .00 | .01 | .14 | .16 | -.22 | .07 |
|  | (-.14, .15) | (-.13, .16) | (-.00, .28) | (.02, .29) | (-.35, -.09) | (-.07, .21) |
|  |  |  |  |  |  |  |
| Trust | -.12 | -.19 | .02 | -.02 | .15 | .05 |
|  | (-.25, .03) | (-.33, -.05) | (-.12, .16) | (-.16, .12) | (.01, .28) | (-.09, .19) |
|  |  |  |  |  |  |  |
| Altruism | .02 | -.06 | -.28 | -.15 | .15 | .22 |
|  | (-.12, .17) | (-.20, .09) | (-.40, -.14) | (-.28, -.01) | (.02, .29) | (.09, .35) |
|  |  |  |  |  |  |  |

*Anxious = Anxious attachment style; Avoidant = Avoidant attachment style; RWA = Right-wing authoritarianism; SDO = Social Dominance Orientation*

**Table A6: rA (below diagonal) and rE (above diagonal) with 95% CI**

|  | Anxious | Avoidant | RWA | SDO | Trust | Altruism |
| --- | --- | --- | --- | --- | --- | --- |
| Anxious |  | 0.41  (.32, .49) | 0.02  (-.07, .12) | 0.02  (-.8, .1) | -0.11  (-0.21, -.0) | -0.06  (-.16, .03) |
| Avoidant | 0.62  (.47, .75) |  | 0.03  (-.06, .12) | -0.01  (-0.1, .09) | 0.02  (-.07, .12) | -0.05  (-.15, .05) |
| RWA | 0.21  (.05, .4) | 0.06  (-.11, .22) |  | 0.13  (.04, .22) | -0.12  (-.21, -.02) | 0.00  (-.1,.1) |
| SDO | 0.12  (-.08, .33) | 0.29  (.08, .5) | 0.74  (.58, .96) |  | -0.07  (-.16, .02) | 0.00  (-.1, .1) |
| Trust | -0.42  (-.63, -.21) | -0.66  (-.37, .0) | -0.45  (-.68, -.29) | -0.56  (-.78, -.36) |  | 0.05  (-.04, .14) |
| Altruism | -0.07  (-.24, 0.1) | -0.19  (-.38, .0) | -0.44  (-.66, -.28) | -0.35  -.55, -0.17 | 0.32  (.14, .51) |  |

*Anxious = Anxious attachment style; Avoidant = Avoidant attachment style; RWA = Right-wing authoritarianism; SDO = Social Dominance Orientation*

**Table A7. Variance components of RWA, with variation shared and unshared with attachment variance**

| Additive genetic | 0.20 |
| --- | --- |
| Common with Attachment | 0.01 |
| specific to RWA | 0.19 |
| Unique environmental variance | 0.27 |
| Common with attachment | 0.00 |
| Specific | 0.27 |
| Specific shared environmental variance | 0.23 |

*Note: estimates derived from best-fitting model (see model four in table 5). RWA = Right-wing authoritarianism*

**Table A8. Variance components of SDO, with genetic and environmental variation shared and unshared with attachment variance**

| Additive genetic | 0.28 |
| --- | --- |
| Common with Attachment | 0.00 |
| Specific to SDO | 0.28 |
| Unique environmental variance | 0.60 |
| Common with attachment | 0.00 |
| Specific to SDO | 0.60 |

*Note: estimates derived from best-fitting model (see model four in table 5), but here SDO and RWA have changed order in the Cholesky model. SDO = Social Dominance Orientation*
